# Endogenous *c-Myc* is essential for p53-induced apoptosis in response to DNA damage *in vivo*

**DOI:** 10.1038/cdd.2014.15

**Published:** 2014-02-28

**Authors:** T J Phesse, K B Myant, A M Cole, R A Ridgway, H Pearson, V Muncan, G R van den Brink, K H Vousden, R Sears, L T Vassilev, A R Clarke, O J Sansom

**Affiliations:** 1 School of Biosciences, University of Cardiff.CF10 3US, Cardiff, UK; 2 Ludwig Institute for Cancer Research, Melbourne, Australia; 3 The Walter and Eliza Hall Institute for Medical Research, Melbourne, Australia; 4 Department of Medical Biology, University of Melbourne, Melbourne, Australia; 5 Beatson Institute for Cancer Research, Glasgow, UK; 6 Department of Gastroenterology & Hepatology, Leiden University Medical Center, Leiden, The Netherlands; 7 Department of Molecular and Medical Genetics, Oregon Health and Science University, Portland, OR, USA; 8 Discovery Oncology, Roche Research Center, Nutley, NJ, USA

**Keywords:** MYC, DNA damage, apoptosis, P53, MDM2, *in vivo*

## Abstract

Recent studies have suggested that C-MYC may be an excellent therapeutic cancer target and a number of new agents targeting C-MYC are in preclinical development. Given most therapeutic regimes would combine C-MYC inhibition with genotoxic damage, it is important to assess the importance of C-MYC function for DNA damage signalling *in vivo*. In this study, we have conditionally deleted the *c-Myc* gene in the adult murine intestine and investigated the apoptotic response of intestinal enterocytes to DNA damage. Remarkably, *c-Myc* deletion completely abrogated the immediate wave of apoptosis following both ionizing irradiation and cisplatin treatment, recapitulating the phenotype of *p53* deficiency in the intestine. Consistent with this, *c-Myc*-deficient intestinal enterocytes did not upregulate *p53*. Mechanistically, this was linked to an upregulation of the E3 Ubiquitin ligase Mdm2, which targets p53 for degradation in c-Myc-deficient intestinal enterocytes. Further, low level overexpression of *c-Myc*, which does not impact on basal levels of apoptosis, elicited sustained apoptosis in response to DNA damage, suggesting c-Myc activity acts as a crucial cell survival rheostat following DNA damage. We also identify the importance of MYC during DNA damage-induced apoptosis in several other tissues, including the thymus and spleen, using systemic deletion of *c-Myc* throughout the adult mouse. Together, we have elucidated for the first time *in vivo* an essential role for endogenous c-Myc in signalling DNA damage-induced apoptosis through the control of the p53 tumour suppressor protein.

A well-known function of the c-Myc protein is its ability to drive apoptosis in numerous cellular contexts.^1, 2, 3^ Of the studies performed, most have concentrated on the ability of c-Myc overexpression to drive apoptosis, unless accompanied by other mutations such as *p53* loss.^4^ The suppression of apoptosis is thought to be a key factor in driving tumorigenesis *in vivo*, for example overexpression of c-Myc in pancreatic islets alone does not induce tumorigenesis unless apoptosis is blocked, for example, by *p53* loss, Bcl-xl overexpression or *ARF* knockout.^5, 6^ The studies examining combined c-Myc overexpression and *p53* loss have implicated p53 directly downstream of c-Myc, but whether this is a direct transcriptional control or indirect (e.g., through c-Myc induction of the DNA damage response) is still controversial.^4^ The most cited model linking c-Myc overexpression to the p53 pathway is via transcription induction of *ARF* by c-Myc, which in turn inhibits Mdm2 (a key negative regulator of p53).^7, 8^ Indeed, in mouse, overexpressing high levels of *c-Myc-ER* from the *Rosa26* locus showed that c-Myc induced apoptosis only in the colon. This was due to higher expression of the *Rosa26* locus and hence overexpression of *c-Myc-ER* within the colon compared with other tissues, leading to the induction of ARF and apoptosis. Genetic deletion of *Arf* rescued this c-Myc-induced apoptosis.^9^

The importance of c-Myc in signalling apoptosis following DNA damage is poorly understood. Thus far, no study has examined this *in vivo,* although *in vitro* studies have suggested it may be of vital importance. There are a number of lines of evidence for this; first (and most importantly) Seoane *et al.*^10^ have shown that in colorectal cancer cell lines depletion of C-MYC reduces apoptosis as a consequence of altering the balance of downstream effectors of P53 signalling. Thus, in the absence of C-MYC, there are increased levels of the antiapoptotic cell cycle arrest protein P21 (a target of P53 which is also transcriptional repressed by C-MYC in a complex with MIZ) and reduced levels of pro-apoptotic genes such as *BAX,* resulting in cell cycle arrest rather than apoptosis. Second, numerous C-MYC transcriptional targets (either activated or repressed by C-MYC) such as *BAX, GADD45A* and *ONZIN* have been shown to be crucial for DNA damage signalling *in vitro*.^11, 12, 13^ Third, c-Myc has been shown to augment apoptosis in fibroblasts following gamma irradiation.^14^ Finally, the post transcriptional regulation of *c-Myc* through *mir34* family members has been suggested to directly impinge on c-Myc function and the cells response to cytotoxic agents.^15^

With the promise of emerging cancer therapies to target C-MYC *in vivo* for the first time using BET inhibitors, there is an urgent requirement to fully understand the role of C-MYC in response to DNA damage. Studies by the Evan group have shown that inhibiting endogenous murine c-Myc *in vivo* through an inducible dominant negative c-Myc protein (OMOMYC) causes regression of a variety of murine tumours (including lung tumours induced by Kras mutation and pancreatic neuroendocrine tumours in mice carrying the *Riptag* transgene).^16, 17^ Moreover, the effects of c-Myc loss were well-tolerated suggesting that c-Myc is an attractive therapeutic target. These data were consistent with our previous studies demonstrating that *c-Myc* was absolutely required for the phenotypes associated with deletion of Apc, although the normal intestine could proliferate without c-Myc (albeit at lower levels).^18^

One of the most tractable systems for studying the DNA damage response *in vivo* is the intestinal crypt. Previously, numerous cytotoxic agents such as cisplatin, ionizing radiation and *N*-methyl-*N*-nitrosourea have been shown to induce apoptosis with a peak induction normally 6–12 h following DNA damage.^19, 20^ This early wave of apoptosis is completely dependent on the nuclear accumulation of p53. The tractability of this system in conjunction with the our previous data showing that *c-Myc* deletion is not immediately deleterious to intestinal enterocytes makes this an ideal system to determine whether c-Myc is important for signalling apoptosis in normal cells following DNA damage.^18^ Importantly, neither of the two studies that conditionally deleted *c-Myc* from the normal intestine has seen any changes in the physiological levels of apoptosis, which could have possibly confounded any analysis.^18, 21^ Both studies showed that *c-Myc*-deficient enterocytes could proliferate; however, our study showed that both the level of proliferation and cell size were reduced compared with wild-type intestinal enterocytes.

In this study, we show that c-Myc is essential for the induction of apoptosis within the intestinal crypt due to the inability of c-Myc-deficient cells to efficiently upregulate p53. Mechanistically, this is associated with high levels of Mdm2 in MYC-deficient cells, and treatment with the MDM2 inhibitor Nutlin restored the stabilization of p53 and induced apoptosis. Importantly, subtle deregulation of c-Myc also has a marked impact on the apoptotic response following DNA damage but no effect on the normal intestine. We also find that other radio-sensitive tissues show a dependence on c-Myc for DNA damage-induced apoptosis. Thus, we propose a general requirement for *c-Myc* expression in making cells permissive to DNA damage-induced apoptosis *in vivo.*

## Results

### *c-Myc*-deficient crypts do not undergo apoptosis following treatment with DNA-damaging agents

We first examined whether *c-Myc* deletion could alter the DNA damage response to ionizing radiation. To induce Cre-mediated gene deletion, *AhCre*^+^ *Myc*^*fl/fl*^ mice and control *AhCre*^+^ *Myc*^+/+^ mice were given three injections IP of 80 mg/kg *β*-naphthoflavone within a single day. This protocol leads to near constitutive levels of *c-Myc* deletion from the intestinal epithelium 4 days following Cre induction^18^ (Supplementary Figure 1a). At this stage, no Cre recombinase expression can be detected (gene loss remains as the deletion event occurs within the stem cell population at the base of the crypts).^22^ *AhCre*^+^ *Myc*^*fl/fl*^ mice and control *AhCre*^+^ *Myc*^+/+^ mice were then exposed to 14 Gray (Gy) of gamma irradiation, and the induction of apoptosis was scored 6 h following the irradiation.

**Figure 1:**
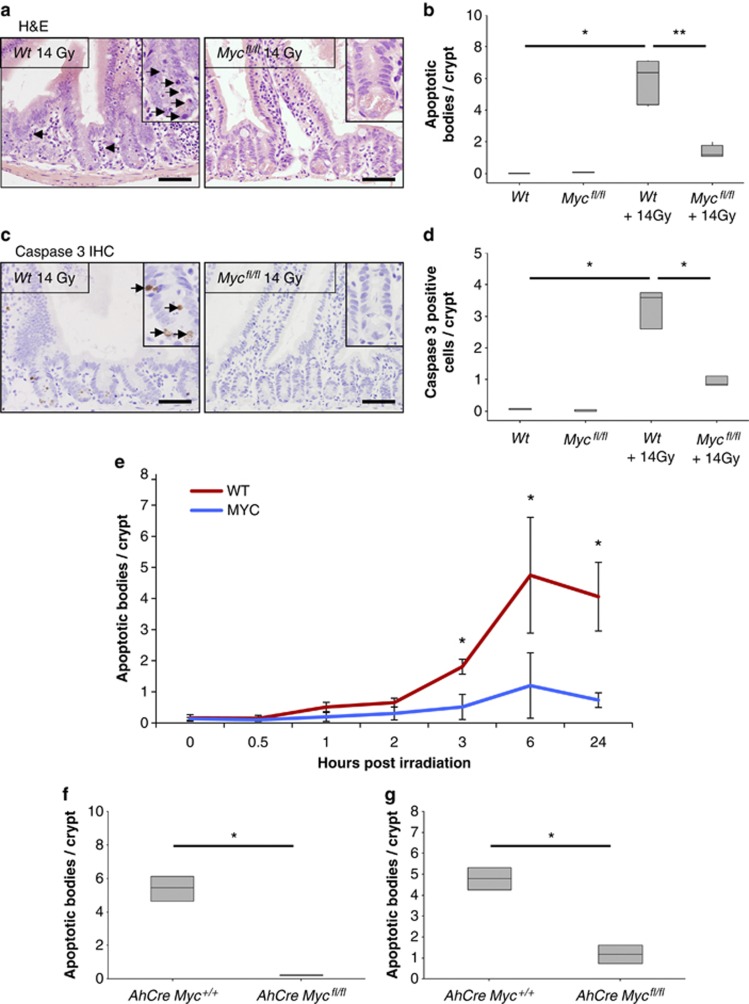
MYC-deficient crypts do not undergo apoptosis following treatment with DNA-damaging agents. (**a**) H&E staining of wild type (*AhCre*^+^ *Myc*^+/+^) and MYC deficient (*AhCre*^+^ *Myc*^*fl/fl*^) intestines 6 h following 14 Gy irradiation, arrows show apoptotic figures in wild-type mice. Scale bars=50 *μ*m. (**b**) Scoring of apoptotic figures from H&E sections shows a significant decrease in apoptosis in MYC-deficient mice following 14 Gy irradiation compared with wild type (* Wt *versus* Wt + 14 Gy, *P* =0.04, Mann Whitney *n*=3 *versus* 6, ** Wt + 14 Gy *versus* Myc + 14 Gy, *P* =0.0041, Mann Whitney *n*=6 *versus* 5). (**c**) Immunohistochemical staining for cleaved (‘active') Caspase 3 was performed on intestinal sections of wild type and MYC-deficient mice. Scale bars=50 *μ*m. (**d**) Quantification of these sections revealed a significant decrease in the number of Caspase-3-positive cells in MYC-deficient mice following 14 Gy irradiation compared with wild type (* Wt *versus* Wt +14 Gy and Wt + 14 Gy *versus* Myc + 14 Gy, *P*=0.04, Mann Whitney *n*=3) (**e**) Graph showing that MYC is essential for the induction of apoptosis following 14 Gy irradiation. Each time point represents at least three mice, illustrating significantly lower levels of apoptosis in MYC-deficient mice at all time points after 2 h (* wt *versus* Myc, *P*=0.04, Mann Whitney *n*=3. Error bars are S.D.). (**f**) Scoring of apoptotic figures from H&E sections shows a significant decrease in apoptosis in MYC-deficient mice compared with wild type following lower levels of irradiation (5 Gy) (* wt *versus* Myc, *P*=0.04, Mann Whitney *n*=3). (**g**) Scoring of apoptotic figures from H&E sections shows a significant decrease in apoptosis in MYC-deficient mice following 10 mg/kg cisplatin treatment compared with wild type (* wt *versus* Myc, *P*=0.04, Mann Whitney *n*=3)

As has previously been reported, H&E analysis of wild-type mice showed a clear induction of apoptosis following 14 Gy gamma irradiation compared with their nonirradiated littermates (Figures 1a and b).^19, 20^ However, the number of apoptotic figures was significantly reduced in c-Myc-deficient crypts given the same dose of irradiation (Figures 1a and b). Indeed, the level of apoptosis in these crypts was closer to nonirradiated controls than irradiated wild-type crypts (Figure 1b). The reduction in apoptosis was not explained due to c-Myc-deficient crypts having fewer cells than wild type,^18^ as we also observed a decrease in the percentage of crypt cells undergoing apoptosis upon *c-Myc* deletion (Supplementary Figure 1b). To confirm the scoring of apoptosis on H&E sections, immunohistochemistry (IHC) was performed against cleaved (‘active') caspase 3,^23^ and once again the number of caspase 3-positive cells was significantly lower in irradiated c-Myc-deficient crypts when compared with wild-type mice given the same dose of gamma irradiation (14 Gy) (Figures 1c and d). To extend this analysis beyond a single time point, we next scored apoptosis at a series of different times following gamma irradiation and found significantly lower levels of apoptosis at all time points subsequent to 2 h in c-Myc-deficient intestinal crypts (Figure 1e).

The resistance to apoptosis of c-Myc-deficient enterocytes was not restricted to high doses of *γ* irradiation, as apoptosis following either a lower dose of irradiation (5 Gy) or cisplatin treatment was also found to be c-Myc dependent (Figures 1f and g). This failure to undergo apoptosis was not simply because c-Myc-deficient cells were not cycling, as we and others have previously shown that c-Myc-deficient intestinal enterocytes can undergo proliferation.^18^

### *c-Myc*-deficient enterocytes do not upregulate *p53*

**Figure 2:**
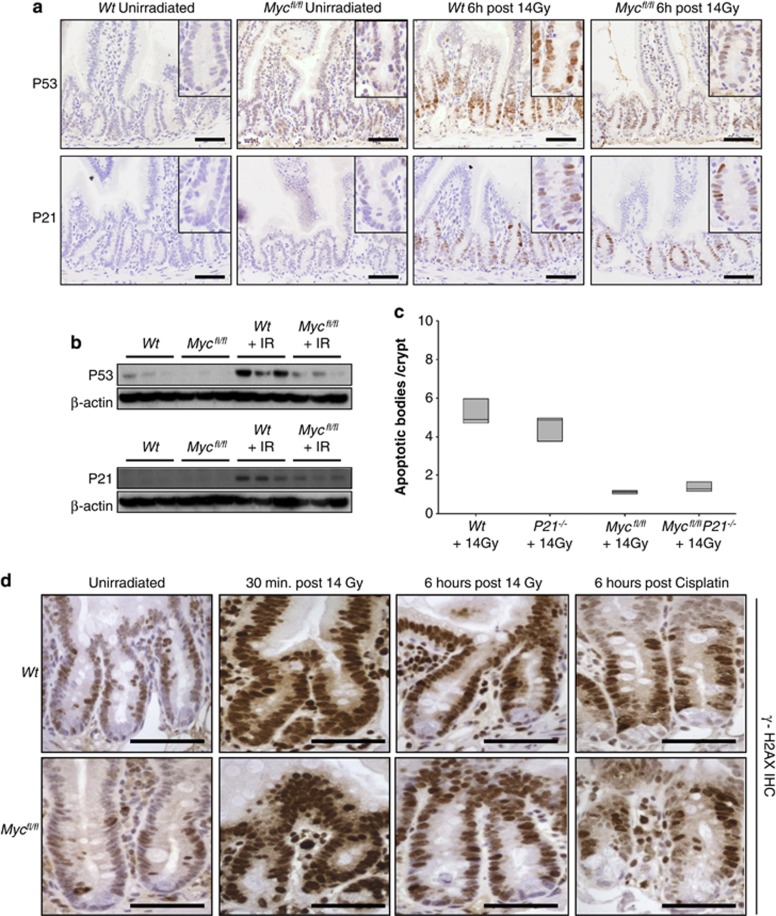
*Myc* deletion prevents P53 accumulation after DNA damage. (**a**) IHC for P53 or P21 in wildtype *(AhCre Myc+/+)* or MYC-deficient (*AhCre Myc*^*fl/f*^) mice 6 h after 14 Gy *γ*-irradiation. Note the induction of nuclear P53 following irradiation in wild-type mice that is attenuated in MYC-deficient enterocytes, and the upregulation of P21 following irradiation in both wild type and MYC-deficient enterocytes. Scale bars=50 *μ*m. (**b**) Immunoblotting shows a marked increase in P53 levels following irradiation that is not seen in MYC-deficient intestinal extracts (top panels). Immunoblotting shows a marked increase in P21 levels following irradiation in both wild type and MYC-deficient intestinal extracts (bottom panels). (**c**) Scoring of apoptotic cells per crypt on the genotypes indicated 6 h after exposure to 14 Gy. *AhCre*^+^ *Myc*^*fl/fl*^ *P21*^*−/−*^ mice display the same lack of apoptotic response to 14 Gy irradiation as the MYC-deficient mice, illustrating that the induction of P21 in *AhCre Myc*^*fl/f*^ mice is not responsible for the failure to upregulate P53 and induce apoptosis (*AhCre Myc*^*fl/fl*^ *versus AhCre*^+^ *Myc*^*fl/fl*^ *P21*^*−/−*^, *P*=0.7656, Mann Whitney *n*=5 *versus* 3). (**d**) Immunohistochemistry for *γ*-H2AX in wild-type *(AhCre Myc+/+)* or MYC-deficient (*AhCre Myc*^*fl/f*^) mice following 14 Gy *γ*-irradiation or cisplatin treatment. The large upregulation of *γ*-H2AX 30 min after irradiation is beginning to clear by 6 h after irradiation as DNA damage is repaired. This expression pattern is observed in both wild type and MYC-deficient crypts (and cisplatin treated), illustrating that MYC-deficient enterocytes are able to sense DNA damage stimuli. Scale bars=50 *μ*m

The phenotype of *c-Myc* deletion paralleled the well-established phenotype of p53 deficiency in the intestine, namely a strong suppression of the immediate wave of apoptosis.^20, 24, 25^ Taking this information with previous data suggesting direct links between c-Myc and p53, we investigated the kinetics of p53 induction in the murine small intestine.^3^ Using immunohistochemistry and western blot, we observed a sharp rise in p53 levels following gamma irradiation that is attenuated in *c-Myc*-deficient crypts (Figures 2a and b and Supplementary Figure 2a). Therefore, this provided a ready mechanism for the abrogated apoptosis.

An alternative mechanism for failed apoptosis is that deletion of *c-Myc* causes a derepression of p21, which could also block apoptosis in this system.^10, 26^ We and others have previously shown that c-Myc deficiency alone is not sufficient to trigger p21 upregulation in the intestine, however, following combined *Apc* and *c-Myc* deletion a clear induction of p21 was observed.^18, 27, 28^ Following irradiation (despite the reduced p53 activation), p21 was still upregulated in *c-Myc*-deficient intestinal enterocytes (Figures 2a and b and Supplementary Figure 2b). To determine whether the lack of transcriptional repression of *p21* by c-Myc was sufficient to block apoptosis, we intercrossed *p21* knockout mice to mice carrying the *Ah Cre* transgene and loxP-flanked *Myc* alleles to generate *AhCre*^+^ *Myc*^*fl/fl*^ *P21*^*−/−*^ mice. Cre was induced in these mice as described above, and these mice were irradiated 4 days after Cre induction and apoptosis was scored 6 h following 14 Gy irradiation. Importantly, *AhCre*^+^ *Myc*^*fl/fl*^ *P21*^*−/−*^ mice displayed the same lack of apoptosis in response to irradiation as the single *c-Myc*-deficient intestinal crypts (Figure 2c). Therefore, this demonstrates that the induction of *p21* following gamma irradiation was not responsible for the block of apoptosis in *c-Myc*-deficient enterocytes and that reduced p53 upregulation was the most likely cause of abrogated apoptosis.

### *c-Myc*-deficient enterocytes sense the DNA damage stimuli

From the literature, there are numerous potential mechanisms that could explain the failure to see increased levels of p53 protein in *c-Myc*-deficient enterocytes. These include failure to detect DNA damage, reduced p53 protein stability or reduced transcription or translation of *p53* mRNA.^3^ Therefore, we decided to test a number of these potential mechanisms.

First, we investigated whether DNA damage recognition was functioning in *c-Myc*-deficient intestinal enterocytes and examined whether there was efficient activation of the DNA damage sensing proteins *γ*H2AX and ATM. This is particularly important as it has previously been suggested that ATM is required for c-Myc to activate p53.^29^ H2AX becomes phosphorylated by ATM at the sites of double-strand breaks in DNA and is essential for their recognition and repair.^30^ We performed immunohistochemistry using an antibody that specifically recognises the activated, phosphorylated form of H2AX (*γ*H2AX) to determine whether this DNA damage response was still intact in *c-Myc*-deficient mice. In wild-type mice, the level of *γ*H2AX is markedly increased 30 min after irradiation, and this level decreases 6 h later as DNA damage is repaired. The activation of *γ*H2AX following either *γ*-irradiation or cisplatin was also observed in MYC-deficient intestinal enterocytes (Figure 2d). We also used an antibody specific to the activated, serine 1981 phosphorylated form of ATM. ATM is a regulator of cellular response to DNA damage, and is auto phosphorylated and associates with other proteins such as p53, Mdm2 and Chk2 to arrest cell cycle at G1. In common with the *γ*H2AX results, ATM is still phosphorylated on serine 1981 in response to DNA damage in wild-type and *c-Myc*-deficient mice following irradiation or cisplatin treatment (Supplementary Figure 2c). Moreover, serine 345 phosphorylation of Chk1 that occurs downstream of ATM and Chk2 was observed at equivalent levels in wild-type and *c-Myc*-deficient intestines following irradiation or cisplatin treatment (Supplementary Figure 2d). Taken together, these results demonstrate that the DNA damage response is still intact in *c-Myc*-deficient enterocytes and suggests that the mechanism behind the failure to induce apoptosis in response to DNA damage is through the control of p53 levels.

### Mdm2 upregulation in *c-Myc*-deficient enterocytes stops p53 accumulation and apoptosis following DNA damage

Given the number of studies in the literature that link c-Myc to control of p53 stability, we next investigated the Mdm2-p53 pathway.^7, 8^ One of the key regulators of p53 protein stability is the Mdm2 E3 ubiquitin ligase. Loss of *Mdm2 in vivo* leads to embryonic death due to the activation of high levels of p53, which can be rescued by co-deletion of *p53*.^31, 32^

**Figure 3:**
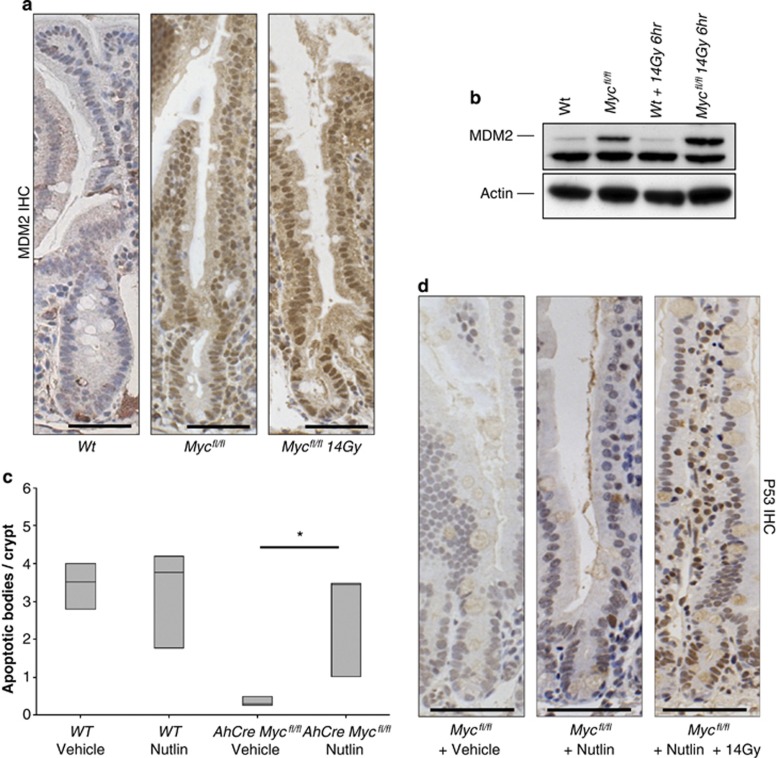
*Myc* deletion causes an accumulation of MDM2. (**a**) MDM2 IHC showing no expression in intestinal crypts of wild type *(AhCre Myc*^+/+^) and a large increase of MDM2 expression in MYC-deficient (*AhCre Myc*^*fl/fl*^) mice in both nonirradiated and 14 Gy irradiated mice. Scale bars=25 *μ*m. (**b**) Immunoblotting for MDM2 shows a marked increase in MDM2 protein levels in both nonirradiated and 14 Gy irradiated mice MYC-deficient intestines. (**c**) MYC-deficient mice treated with Nutlin exhibited a full restoration of the apoptotic response (* wt *versus* Myc, *P* =0.04, Mann Whitney *n*=3), which was not observed in MYC-deficient mice treated with vehicle. Note this restoration of apoptosis correlates with an induction of P53 in MYC-deficient mice treated with Nutlin. (**d**) Immunohistochemistry for P53 demonstrating that P53 now accumulates in nutlin-treated MYC-deficient intestines following gamma irradiation but not vehicle-treated or nonirradiated MYC-deficient intestines. Scale bars=50 *μ*m

First we examined the levels of Mdm2 following *c-Myc* deletion by immunohistochemistry and immunoblotting. We found a marked upregulation of Mdm in *c-Myc*-deficient cells that was maintained following irradiation (Figures 3a and b). To test whether this was functionally important for blocking apoptosis in the *c-Myc*-deficient crypt cells, we employed the Mdm2 antagonist, Nutlin-3a. Nutlin is a selective small-molecule inhibitor of the p53-Mdm2 interaction that releases p53 from Mdm2 control, leading to accumulation of the tumour suppressor protein and activation of the P53 pathway.^33, 34^ Treatment of cancer cells with wild-type P53 induces cell arrest and apoptosis *in vitro* and suppresses the growth of human tumour xenografts in nude mice.^33, 35^

*c-Myc*-deficient mice were treated with Nutlin twice daily on days 1–3 post Cre induction and a final time 3 h before 14 Gy irradiation on day 4. This methodology was employed as it has been shown previously that Nutlin can knockdown Mdm2 for approximately 12 h *in vivo*, and a number of doses are required for full functional inhibition.^33^ Apoptosis was scored in WT and *c-Myc*-deficient mice 6 h following irradiation and a restoration of the apoptotic response was observed in *c-Myc*-deficient mice treated with Nutlin, though not vehicle (Figure 3c). Consistent with previous reports, Nutlin treatment had no obvious impact on proliferation or apoptosis of wild-type intestinal enterocytes (either alone or treated with 14 Gy) (data not shown).^35^ Most importantly, this restoration of the apoptotic response correlated with the induction of p53 in the *c-Myc*-deficient mice treated with Nutlin (Figure 3d). These data demonstrate that restoration of p53 function, via Nutlin treatment, is sufficient to rescue the blocked apoptosis phenotype in *c-Myc*-deficient enterocytes and therefore establishes that c-Myc regulates apoptosis via p53 in the mammalian intestine.

### Deregulated overexpression of c-Myc increases and sustains DNA damage-induced apoptosis

Our data demonstrate that MYC loss of function completely abrogates the apoptotic response induced by DNA damage. We next asked if subtle overexpression of c-Myc could influence the DNA damage response. To alter c-Myc levels, we used mice where the *c-Myc* cDNA has been targeted in the *ROSA26* locus and is under the control of Cre expression through Lox Stop Lox elements in the promoter (*ROSA-Floxed-Stop (RFS)-*myc^WT^).^36^ This system permits inducible, relatively low level, deregulated c-Myc overexpression, previously shown to be sufficient to overcome the reduced proliferation in *c-Myc*-deficient intestines.^37^

**Figure 4:**
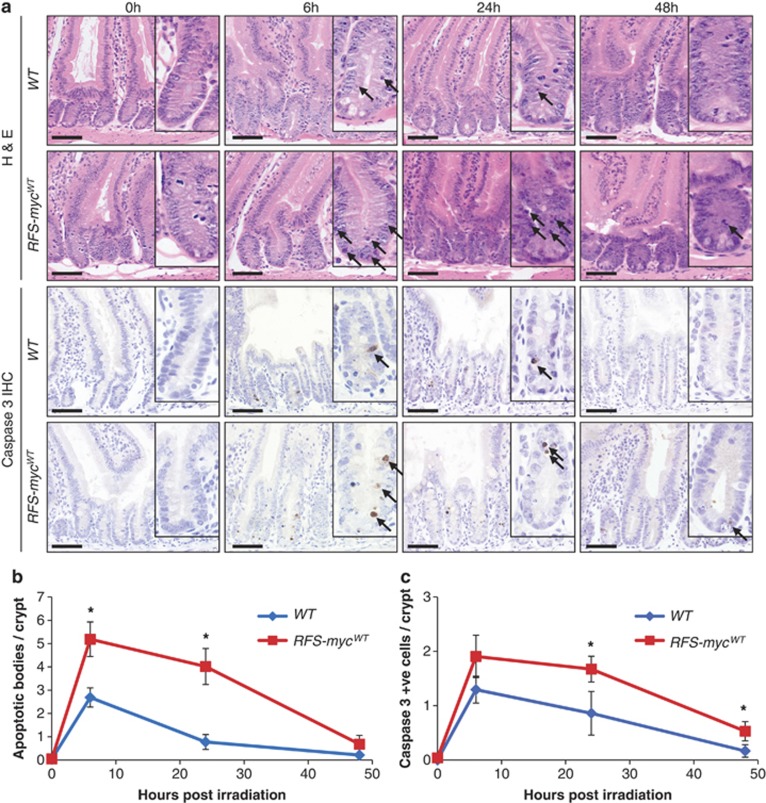
Deregulated MYC expression increases DNA damage-induced apoptotic response. (**a**) H&E staining (top panels) and caspase 3 IHC (bottom panels) of wild type (*AhCre Rosa26*^+/+^) and *Myc* transgene expressing (*RFS-myc*^*WT*^) small intestines 0, 6, 24 and 48 h following 5 Gy irradiation, arrows indicate apoptotic bodies and caspase 3-positive cells. Scale bars=50 *μ*m. (**b**) Scoring of apoptotic bodies from H&E sections shows a significant increase in apoptosis in small intestines overexpressing MYC at 6 h (* WT *versus RFS-myc*^*WT*^, *P*=0.0184, Mann Whitney *n*=5 *versus* 3) and 24 h (* WT *versus RFS-myc*^*WT*^, *P*=0.04, Mann Whitney *n*=3) following 5 Gy irradiation (Error bars are standard deviation). (**c**) Scoring of caspase 3-positive cells shows a significant increase in apoptosis in small intestines overexpressing MYC at 24 h (* WT *versus RFS-myc*^*WT*^, *P*=0.04, Mann Whitney *n*=3) and 48 h (* WT *versus RFS-myc*^*WT*^, *P*=0.04, Mann Whitney *n*=3) following 5 Gy irradiation (Error bars are S.D.)

Following activation of *c-Myc* transgene expression, we induced DNA damage using 5 Gy IR and examined the impact of increasing levels of c-Myc for the apoptotic response. This lower level of DNA damage permits observations over extended time points. Increasing c-Myc levels had no impact on the basal levels of apoptosis (Figures 4a and b, 0 h time point). Following irradiation, however, the peak apoptotic response at 6 h was significantly increased (Figures 4a and b). In addition, whereas in control mice the rate of apoptosis had dropped sharply 24 h after DNA damage, it was maintained at a significantly higher level in intestines expressing deregulated c-Myc (Figures 4a and b). To confirm these observations, immunohistochemistry against active caspase 3 was performed. In line with the histological data, increased c-Myc levels led to increased numbers of caspase 3-positive cells (Figures 4a and c). Thus, in addition to being required for intestinal apoptosis following DNA damage, deregulated c-Myc expression can also elevate and maintain it. These data strongly suggest that the precise regulation of c-Myc levels is important for the kinetics of apoptosis following DNA damage.

### Loss of *c-Myc* throughout the adult mouse prevents DNA damage-induced apoptosis in radiosensitive tissues

**Figure 5:**
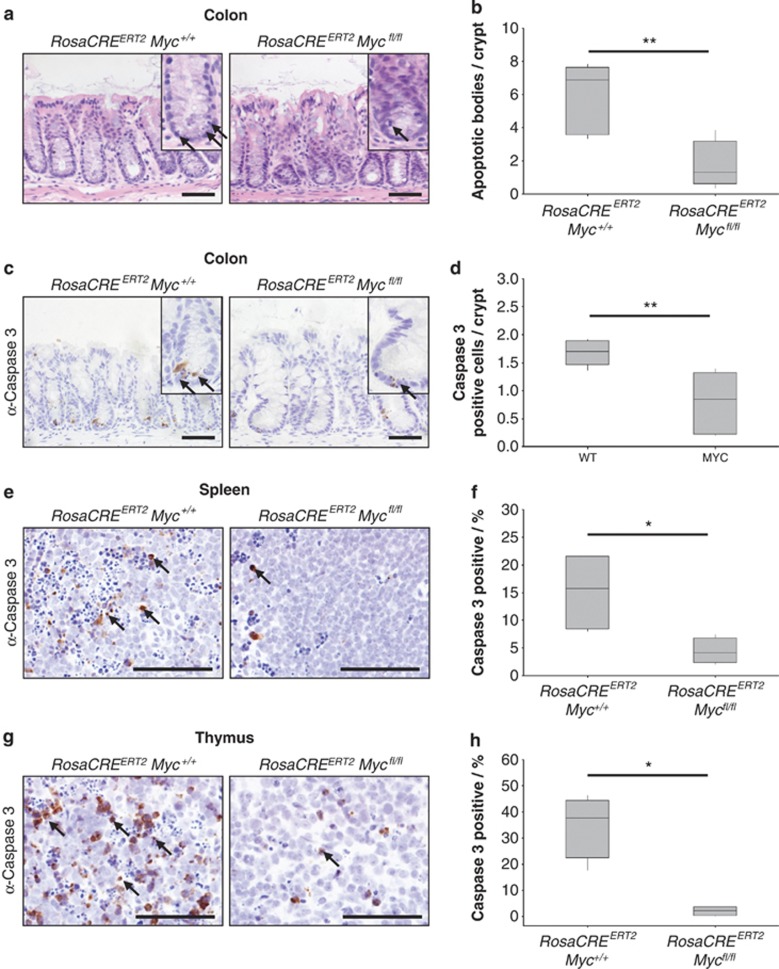
MYC deletion prevents DNA damage-induced apoptosis in multiple tissues. (**a**) H&E staining of wild type (*RosaCre*^*ERT2*^ *Myc*^+/+^) and MYC-deficient (*RosaCre*^*ERT2*^ *Myc*^*fl/fl*^) colons 6 h following 14 Gy irradiation, arrows show apoptotic bodies. Scale bars=50 *μ*m. (**b**) Scoring of apoptotic bodies from H&E sections shows a significant decrease in apoptosis in MYC-deficient colons following 14 Gy irradiation compared with wild type (** wt *versus* Myc, *P*=0.0059, Mann Whitney *n*=5 *versus* 7). (**c**) Caspase 3 IHC staining of wild type (*RosaCre*^*ERT2*^ *Myc*^+/+^) and MYC-deficient (*RosaCre*^*ERT2*^ *Myc*^*fl/fl*^) colons 6 h following 14 Gy irradiation, arrows indicate Caspase 3-positive cells. Scale bars=50 *μ*m. (**d**) Scoring of Caspase 3-positive cells (percentage of total cells: *n*>500) shows a significant decrease in apoptotic cells in MYC-deficient colons (** wt *versus* Myc, *P*=0.0059, Mann Whitney *n*=5*versus*7). (**e**) Caspase 3 IHC staining of wild type (*RosaCre*^*ERT2*^ *Myc*^+/+^) and MYC-deficient (*RosaCre*^*ERT2*^ *Myc*^*fl/fl*^) spleens 6 h following 14 Gy irradiation, arrows indicate Caspase 3-positive cells. Scale bars=50 *μ*m. (**f**) Scoring of Caspase 3-positive cells (percentage of total cells: *n*>500) shows a significant decrease in apoptotic cells in MYC-deficient spleens (* wt *versus* Myc, *P*=0.0152, Mann Whitney *n*=4). (**g**) Caspase 3 IHC staining of wild type (*RosaCre*^*ERT2*^ *Myc*^+/+^) and MYC-deficient (*RosaCre*^*ERT2*^ *Myc*^*fl/fl*^) thymus 6 h following 14 Gy irradiation, arrows indicate Caspase 3-positive cells. Scale bars=50 *μ*m. (**h**) Scoring of Caspase 3-positive cells (percentage of total cells: *n*>500) shows a significant decrease in apoptotic cells in MYC-deficient thymus (* wt *versus* Myc, *P*=0.0152, Mann Whitney *n*=4)

Given therapeutic inhibition of C-MYC would likely suppress its function throughout the body, we next tested if our findings extended beyond the intestinal epithelium. Outside of the small intestine other radiosensitive tissues include the colon, spleen and thymus. Importantly, the adult thymus proliferates at a very low rate, and thus this is an excellent tissue to study the induction of p53-dependent apoptosis that is uncoupled from proliferation. The ubiquitously expressed *RosaCreER* permits conditional gene deletion in multiple tissues. To test recombination rates, we generated *RosaCreER*^+^ *Lox-stop-lox RFP* mice and induced them with tamoxifen. High levels of RFP expression were observed in multiple tissues including the colon, spleen and thymus indicating successful recombination in these tissues (Supplementary Figures 3a–c). To test if c-Myc is a critical mediator of apoptosis in these tissues, we induced control *RosaCreER*^+^ *Myc*^+/+^ and experimental *RosaCreER*^+^ *Myc*^*fl/fl*^ mice, irradiated them with 14 Gy 6 days post induction and killed them 6 h later. In the colonic epithelium, we observed significantly decreased levels of apoptosis (Figures 5a and b) that was coincident with loss of c-Myc protein (Supplementary Figure 3d). As before, we confirmed this observation with caspase 3 staining (Figures 5c and d). Scoring of caspase 3 positivity also indicated a significantly reduced apoptotic response in both the spleen and thymus (Figures 5e and h). Similar to the small intestine, we observed attenuated p53 induction in the spleen and thymus indicating that c-Myc-dependent upregulation of p53 may be responsible for the induction of apoptosis in multiple tissues. (Supplementary Figure 4a and b). Interestingly, despite a failure to induce apoptosis, p53 induction was comparable between WT and *c-Myc*-deficient colonic cells (Supplementary Figure 4c). This indicates that, in the colon, c-Myc either controls apoptosis downstream of p53 signalling or via a P53-independent mechanism. Together, these data demonstrate that c-Myc function is required for DNA damage-induced apoptosis in multiple epithelial and lymphoid tissues, strongly suggesting that it is a general mediator of apoptosis *in vivo.*

## Discussion

**Figure 6:**
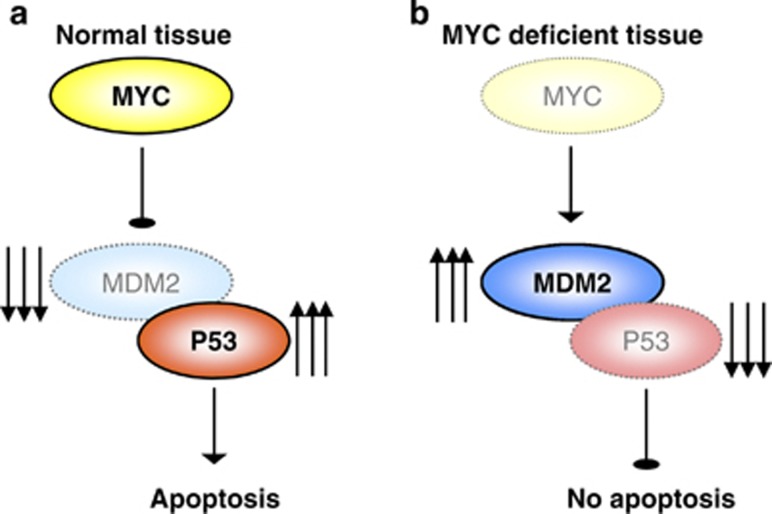
Model of MYC regulation of apoptosis in response to DNA damage in the intestine. (**a**) In wild type, MYC-proficient mice, the intestinal epithelial cells respond to DNA damage by upregulating MYC, which then inhibits MDM2. This allows P53 levels to increase and induce apoptosis. (**b**) When MYC is deleted from the intestinal epithelial cells they can no longer inhibit MDM2 in response to DNA damage, and consequently P53 is not upregulated resulting in reduced apoptosis

Here, we demonstrate for the first time in an *in vivo* setting that endogenous c-Myc is required for efficient induction of apoptosis following DNA damage. We show that this is due to a failure to upregulate p53 owing to increased levels of the E3 ubiquitin ligase Mdm2 (Figure 6). As this mechanism is conserved in multiple tissues, we contend that c-Myc serves as a universal regulator of apoptosis *in vivo*.

c-Myc has the ability to sensitize or induce apoptosis *in vitro*, but its role in this process is not well established *in vivo*. We found that deletion of *c-Myc* throughout the adult mouse strongly suppressed the apoptotic response following DNA damage. *c-Myc-*deficient intestinal epithelial cells are able to proliferate and thymocytes proliferate very slowly, thus the lack of apoptosis is not simply a byproduct of the proliferative function of c-Myc. Indeed, as overexpression of c-Myc in the small intestine increased both the level and length of the apoptotic response, we believe c-Myc should be regarded as an apoptotic permissivity factor. Thus, it is also likely that the precise regulation of c-Myc throughout DNA damage-induced apoptosis is important for the cessation of the apoptotic response following DNA repair. Interestingly, subtle deregulation of c-Myc expression did not lead to increased levels of intestinal apoptosis in the absence of DNA damage. This appears to contrast with previous studies demonstrating that overexpression of c-Myc-ER leads to ectopic apoptosis in the colon.^9^ This may be due to a difference in apoptotic response between the small intestine and colon. Alternatively, it may highlight a difference between overexpression of c-Myc-ER and c-Myc. It is possible that c-Myc-ER accumulates to high levels in the absence of tamoxifen. Thus, when tamoxifen is administered, the acute burst of c-Myc-ER activity induced is sufficient to drive apoptosis. As c-Myc expression is regulated by multiple transcriptional and post transcriptional mechanisms *in vitro*, it is likely that its regulation *in vivo* is also complex.^38^ It is interesting to note that previous studies have shown that reducing the *c-Myc* targeting *mir34b/c* microRNA causes increased DNA damage apoptosis, suggesting physiological regulation of c-Myc levels following DNA damage modifies the apoptotic response.^15^

We observed a failure to induce p53 in *Myc*-deficient intestinal, splenic and thymic cells. In the intestine, this was associated with increased Mdm2 expression and treatment with the Mdm2 inhibitor Nutlin rescued both p53 activation and induction of apoptosis. This suggests that c-Myc regulates apoptosis via post translational regulation of p53 stability *in vivo.* ATM can also activate p53 suggesting that failed DNA damage signalling could also impact on apoptotic response.^29^ Importantly, we found DNA damage signalling intact in *c-Myc*-deficient intestines indicating that control of Mdm2 is the primary mechanism through which c-Myc regulates apoptosis *in vivo*. The precise mechanism linking c-Myc levels to Mdm2 expression is unclear, but Mdm2 has previously been shown to be a direct transcriptional target of NMYC in neuroblastoma.^39^ Although this study demonstrated a role in activating Mdm2 transcription, c-Myc proteins are also well-defined transcriptional repressors so it is tempting to speculate that Mdm2 transcription may be directly inhibited by c-Myc. Alternatively, the c-Myc transcriptional target Nucleolin has been shown to directly bind to and inhibit Mdm2.^40^ This binding was also shown to lead to reduced levels of Mdm2 protein, thus making it an attractive potential mediator of the phenotypes we observe. A thorough dissection of these potential interactions will be important for further understanding of this process.

Interestingly, it does not appear that this mechanism extends to the colon as *c-Myc*-deficient colonic cells efficiently upregulate P53 following irradiation. This indicates that induction of the apoptotic programme, although c-Myc dependent, is fundamentally different in the colon. With regards to this, the transcriptional activation and oligomerization of the apoptosis regulator Bax has been shown to be controlled by c-Myc.^11, 41^ Thus, it is possible that colonic cells engage a different apoptotic programme from those in the intestine. Determining the mechanistic basis for this difference will be an interesting future avenue of research.

C-MYC is one of the most commonly altered genes in human cancer with gene amplifications and transcriptional activation especially common. As such, it is a very attractive therapeutic target and several recent studies have highlighted this potential. In particular, c-Myc inhibition using a dominant negative form of the protein termed OMOMYC was shown to regress tumours from both lung and pancreatic mouse tumour models.^16, 17^ Importantly, although expression of OMOMYC suppressed proliferation in various tissues, these effects were completely reversed upon cessation of treatment. This suggests that C-MYC inhibition would not be overly toxic and is therefore a viable therapeutic target. Inhibition of BET bromodomain proteins has been shown to inhibit C-MYC function and tumorigenesis, thus chemical suppression of MYC may be possible.^42^ As most C-MYC inhibitory agents would be delivered alongside DNA-damaging agents, our findings that c-Myc is required for apoptosis are important. It will be important to determine if our findings extend to tumorigenic cells as they may also be protected from apoptosis by C-MYC inhibition. If this is the case then precise coordination of treatment regimens may be required to achieve maximum functionality. Interestingly, OMOMYC or BET inhibition both lead to proliferation arrest and apoptosis in transformed cells, indicating that they may not have the same requirement for C-MYC during apoptosis. In fact, this may lead to an unexpected benefit of C-MYC inhibition, namely some protection from the side effects of commonly used therapeutics. It will be important to gain further understanding of this process *in vivo* to help better understand this issue.

In conclusion, we have demonstrated for the first time, a requirement for c-Myc function during the induction of apoptosis following DNA damage. We find this function is constant in multiple tissues defining c-Myc as a general mediator of apoptosis *in vivo*.

## Materials and Methods

### Mouse experiments

All experiments were performed under the UK Home Office guidelines. Outbred male mice from 6 to 12 weeks of age were used, which were segregating for the C57BLJ and S129 genomes. The alleles used were as follows: *Myc*^*fl*^, *AhCre*, *P21, ROSA-Floxed-Stop (RFS)-myc*^*WT*^*, RosaCreER* and *ROSA-tdRFP*.^22, 36, 43, 44, 45, 46, 47^ *Myc* experiments were also confirmed on mice that had been backcrossed to C57Bl6J for five generations.

Cre induction was carried out by giving *AhCre*^+^ *Myc*^++^ and *AhCre*^+^*Myc*^*fl/fl*^ 3 intraperitoneal (IP) injections of 80 mg/kg *β*-naphthoflavone in a single day. Mice were then given DNA damage 4 days after induced gene deletion. Previous experiments have shown that, using this protocol, no significant induction of apoptosis is seen in induced (*AhCre*^+^ *Myc*^++^) when compared with uninduced *AhCre*^+^ *Myc*^++^ or induced wild type (mice not carrying the AhCre transgene) at day 4 after induction.

For assessing whether MYC deficiency affects the DNA damage response following gamma irradiation ‘wild type' *AhCre*^+^ *Myc*^++^ and MYC-deficient *AhCre*^+^ *Myc*^*fl/fl*^ mice were irradiated with 14 Gy irradiation using a Cs^137^ source delivered at a dose rate of at 0.423 Gy/min. Mice were then collected at 30 min, 1, 2, 3, 6, 12, 24 and 48 h time points following the irradiation. At least three mice were used for each time point. For cisplatin treatment, mice were given a single IP injection of 10 mg/kg Cisplatin (purchased from David Bull Laboratories (Warwick, UK) and distributed by Faulding Pharmaceuticals).

For assessing whether MDM2 upregulation abrogated apoptosis following *Myc* deletion, ‘wild-type' *AhCre*^+^ *Myc/*^++^ and *Myc*-deficient *AhCre*^+^ *Myc*^*fl/fl*^ mice were gavaged with either 200 *μ*l of vehicle or 200 mg/kg nutlin-3a (synthesized at the Roche Research Center, Nutley, NJ, USA) twice a day as previously described.^33^ On day 4 post Cre induction, mice were given a single application of nutlin and irradiated with 14 Gy and collected 6 h following the irradiation.

### Tissue isolation

Tissue isolation was carried out as follows: the proximal 7 cm was fixed overnight in methacarn (methanol, chloroform and acetic acid; 4 : 2 : 1) and then paraffin embedded. The following 3 cm was preserved in RNA later (Sigma, Gillingham, UK). The following 5 cm was bundled using surgical tape and fixed in 4% formaldehyde at 4 °C for 24 h before processing. The remainder was fixed in methacarn.

### Assaying apoptosis *in vivo*

Apoptotic bodies were scored from H&E sections. Twenty-five full crypts were scored from a minimum of three mice of each genotype. Apoptosis was confirmed by immunohistochemical staining against active caspase 3 (1:750, R&D systems, Abbingdon, UK).

### Immunohistochemistry

Primary antibodies used for immunohistochemistry: P21 (1 : 500, Santa Cruz, Dallas, TX, USA; M19), P53 (1 : 100 MS-104, PAB240 Neomarkers) and P53 (VectorLabs, Peterborough, UK; CM5), CHK1 pS345 (1 : 100, Cell Signalling, Danvers, MA, USA), ATM pS1981 (1 : 500, ROCKLAND 200-301-500), MDM2 (1 : 200, Lab Vision, Waltham, MA, USA; smp14 ms- 291-p1), *γ*-H2AX (1 : 300 Upstate), MYC (1 : 500, Santa Cruz, N-262, sc764).

### Epithelial extractions

To obtain a population of epithelial cells, an epithelial extraction protocol based on Bjerknes & Cheng^48^ was performed. In brief, 10 cm of small intestine was flushed with water before being tied and everted over a glass rod. Vibration was then applied, and the intestine placed in 10 mM EDTA in Hanks' Balanced Salt Solution (HBSS; Gibco, Paisley, UK) at 37 °C for 15 min. The intestine was moved into a fresh tube of 10 mM EDTA/HBSS and incubated for a further 15 min. Epithelial cells were collected by centrifugation (2700 × *g*, 4 °C, 15 min).

### Western blot analysis

Protein was extracted from epithelial extracted samples by standard methods using lysis buffer (20 mM Tris-Hcl pH8.0, 2 mM EDTA (pH8.0), 0.5% (v/v) NP-40) containing protease inhibitors (Complete Mini Protease inhibitor tablets, Roche, Burgess Hill, UK) and phosphatase inhibitors (25 mM sodium *β*-glycerophosphate, 100 mM sodium fluoride, 20 nM Calyculin A, 10 mM sodium pyrophosphate). Solubilised proteins (20 *μ*g) were separated by standard SDS-PAGE on a 10% polyacrylamide separating gel with 5% stacking gel and subsequently transferred to PVDF membrane (Hybond-P, Amersham Biosciences, Buckinghamshire, UK) by standard methods. Primary antibodies and conditions used to probe blots were rabbit anti-MDM2 (1 : 1000; R&D systems AF1244), mouse anti-P53 (1 : 1000; Cell Signalling Technology 1C12), rabbit anti-P21 (1 : 200; Santa Cruz sc479) and mouse anti-*β*-actin (1 : 5000; Sigma). Appropriate HRP-conjugated secondary anti-rabbit or anti-mouse antibodies were used (Amersham Biosciences).

## Abbreviations

NMNU: *N*-methyl-*N*-nitrosourea
BET: bromodomain and extra-terminal
IHC: immunohistochemistry
Gy: gray
