# Supplementary figures and images for: Endogenous c-Myc is essential for p53-induced apoptosis in response to DNA damage in vivo
Source: Cell Death Differ. 2014 Feb 28;21(6):956–66. doi: 10.1038/cdd.2014.15 (PMC4013513; doi:10.1038/cdd.2014.15)

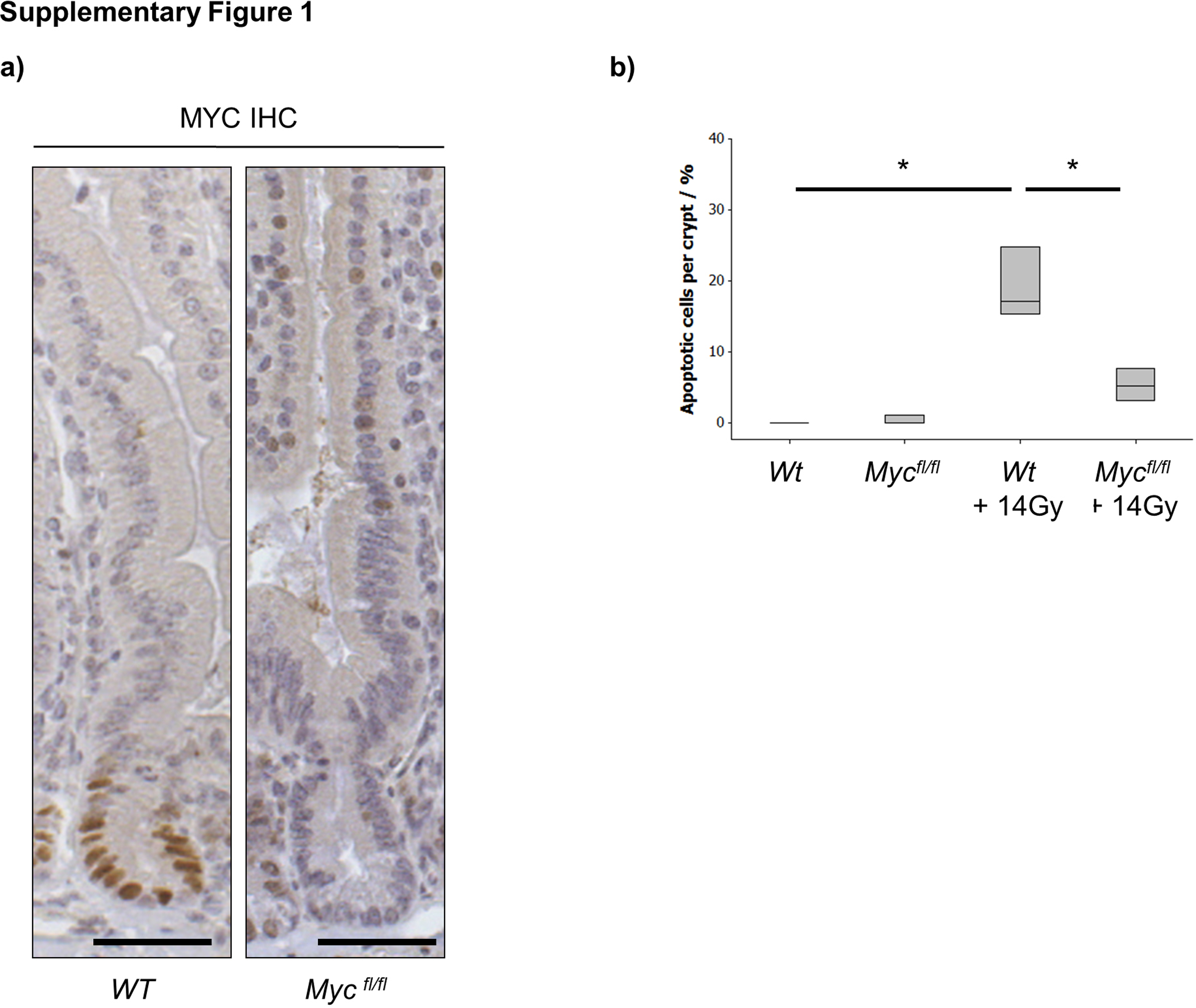

Supplement: Supplementary Figure 1 [file cdd201415x1.tif]

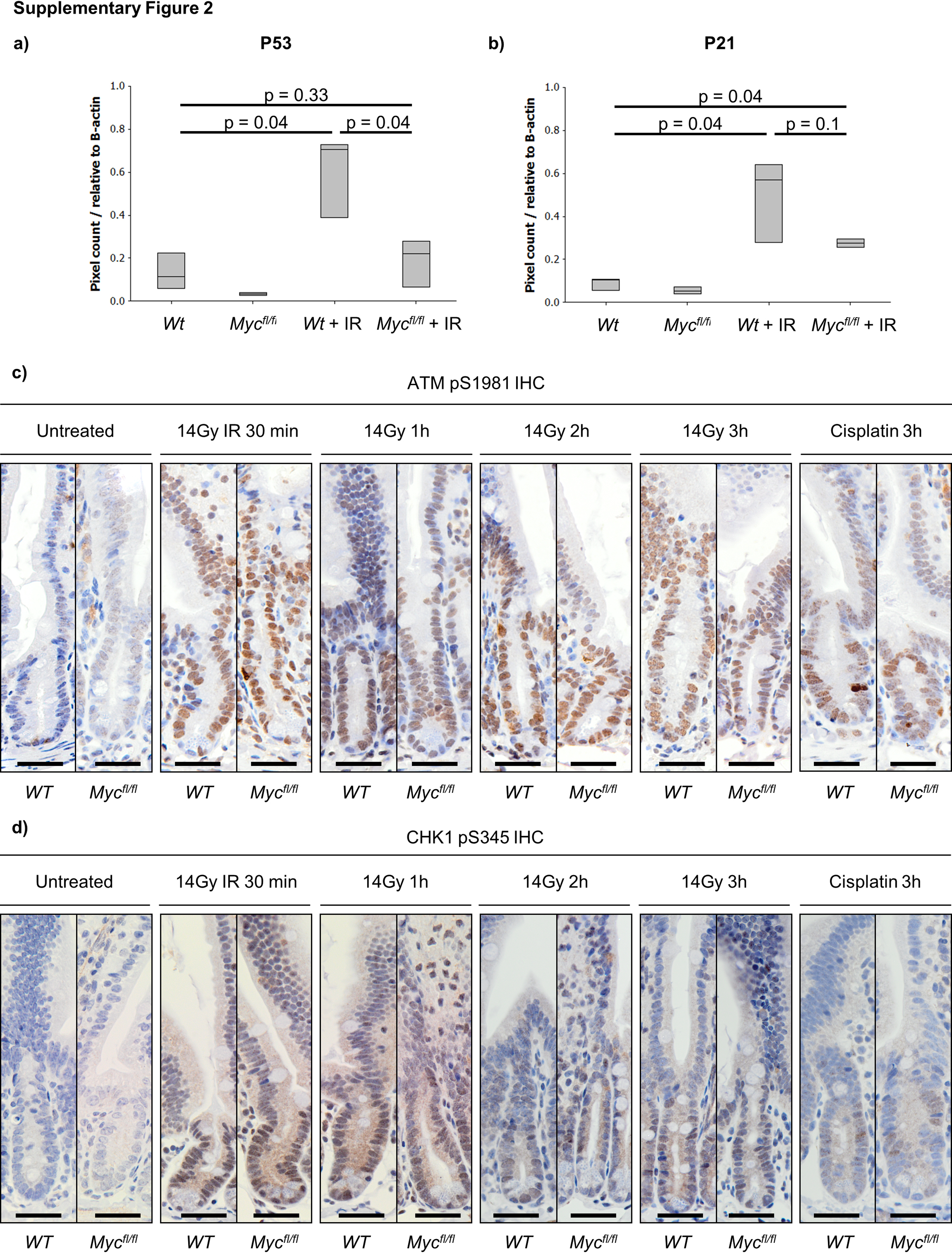

Supplement: Supplementary Figure 2 [file cdd201415x2.tif]

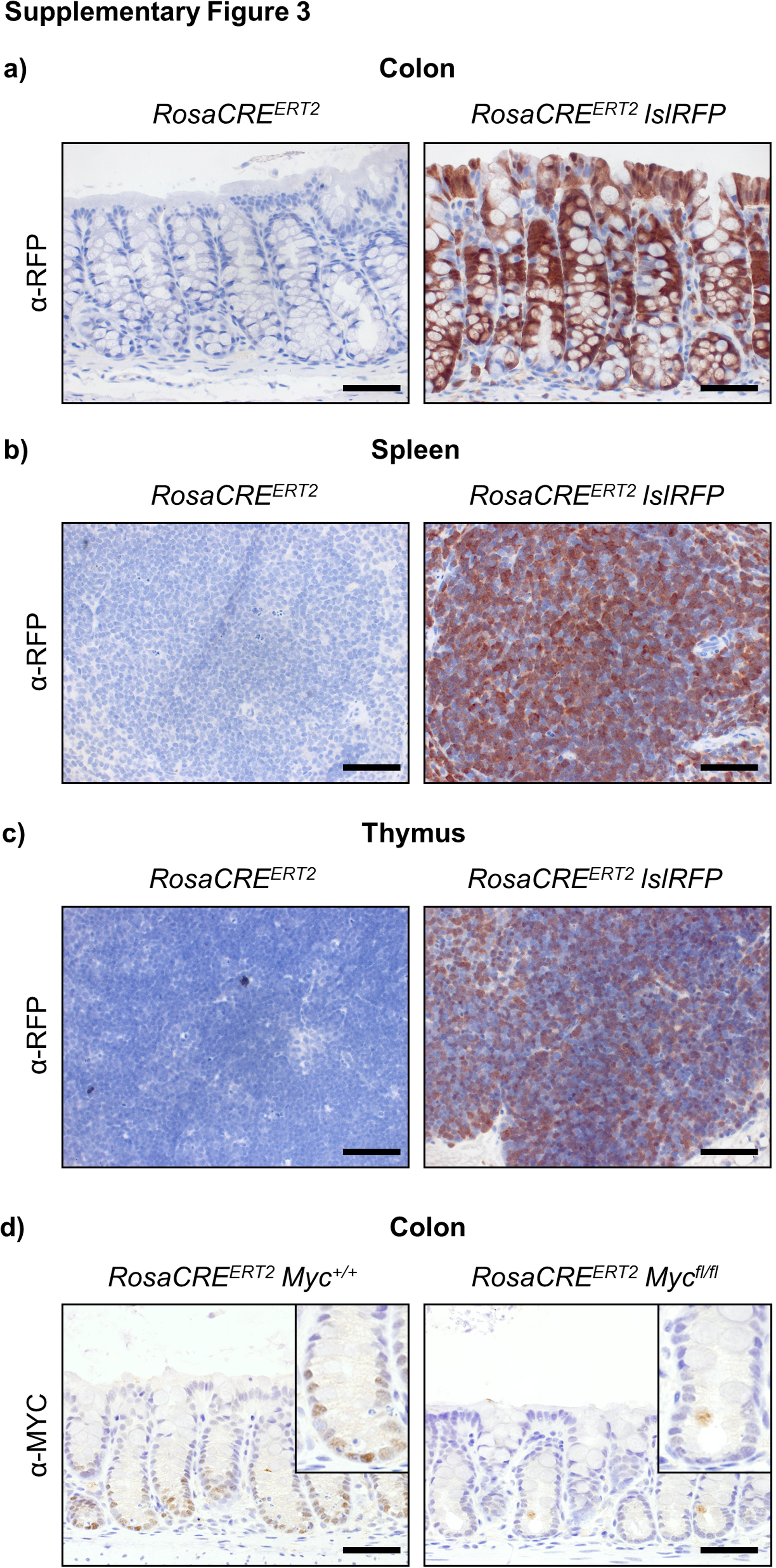

Supplement: Supplementary Figure 3 [file cdd201415x3.tif]

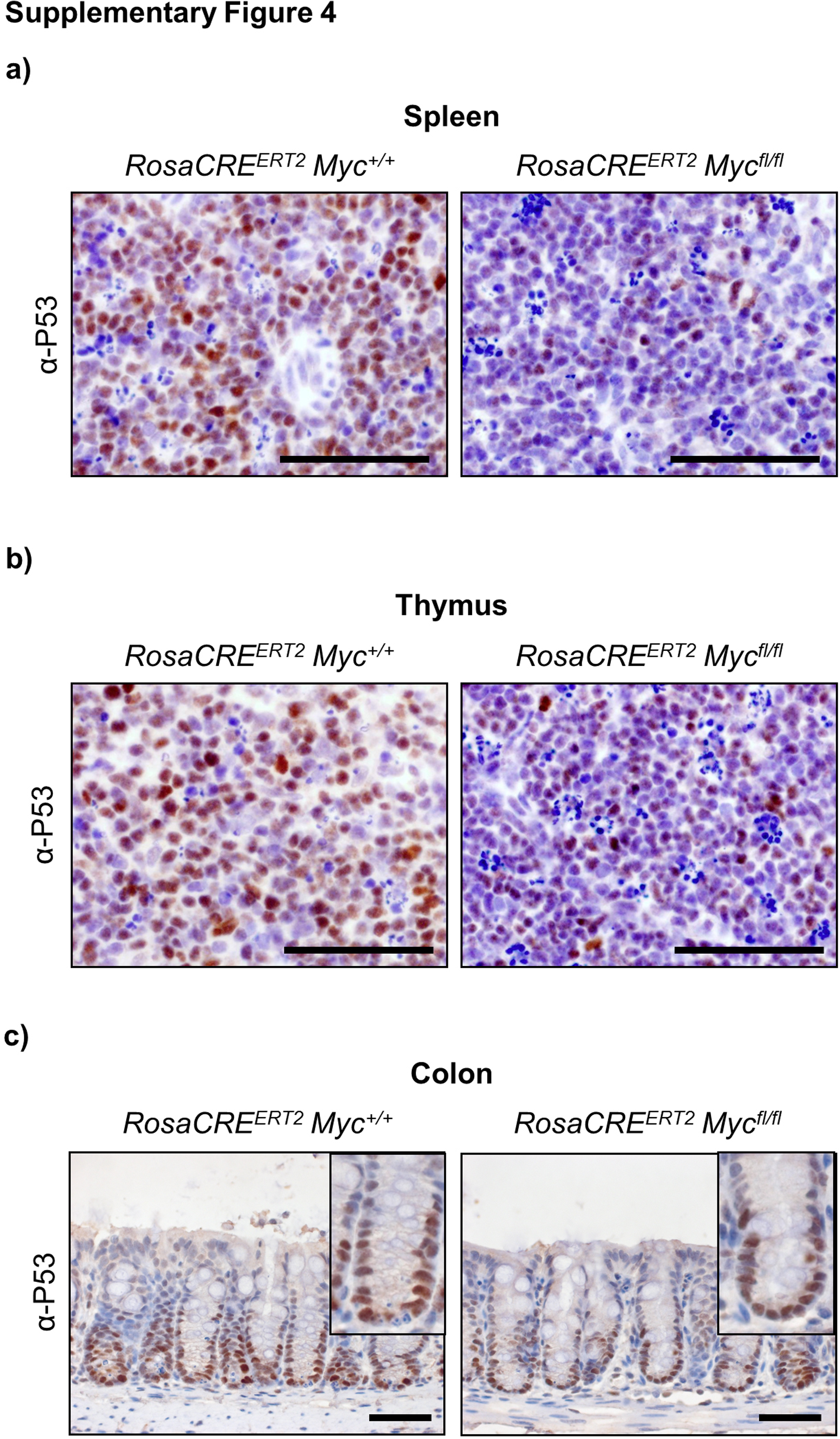

Supplement: Supplementary Figure 4 [file cdd201415x4.tif]
