# Supplementary material for: Endogenous c-Myc is essential for p53-induced apoptosis in response to DNA damage in vivo
Source: Cell Death Differ. 2014 Feb 28;21(6):956–66. doi: 10.1038/cdd.2014.15 (PMC4013513; doi:10.1038/cdd.2014.15)
Supplement: Supplementary Figure Legends [file cdd201415x5.doc]

**Supplementary Data Includes:**

**4 Supplementary Figures – 1 pdf**

**4 Supplementary Figure Legends – This file**

**Supplementary Figure 1: Efficient Myc deletion 4 days following Cre induction.**

**(a)** Myc immunohistochemistry performed on intestinal sections from wild type (*AhCre^+^ Myc^+/+^*) and Myc deficient (*AhCre^+^ Myc^fl/fl^ )* mice. Note complete loss of Myc protein from the intestinal crypt of *AhCre^+^ Myc^fl/fl^* mice. Scale bars = 25µm. **(b)** Scoring of apoptotic figures from H&E sections as a percentage of total crypt cells, at least 100 cells were scored for each genotype / treatment. This shows a significant decrease in apoptosis in MYC deficient mice following 14Gy irradiation compared to wild type (* Wt vs Wt + 14Gy, p = 0.04, Mann Whitney n=3, * Wt + 14Gy vs Myc + 14Gy, p = 0.04, Mann Whitney n=3).

**Supplementary Figure 2: ATM and CHK1 are still activated in response to DNA damage in wild type and Myc deficient mice. (a)** Quantification of P53 immunoblot band intensities relative to β-actin loading control. Note the significant induction of P53 expression following irradiation that is dependent on Myc. **(b)** Quantification of P21 immunoblot band intensities relative to β-actin loading control. Note the significant induction of P21 expression following irradiation in both Wt and Myc deficient tissue. **(c)** IHC for ATM pS1981 showing low levels of expression in the intestinal crypts of wild type*, AhCre Myc+/+,* (Wt) and Myc deficient*, AhCre Myc^fl/fl^* mice, and equal levels of upregulation in wild type and Myc deficient mice, 0.5 - 3 hours following 14Gy irradiation or cisplatin treatment. Scale bars = 25µm. (**d)** IHC for CHK1 pS345 showing low levels of expression in the intestinal crypts of wild type and MYC deficient mice and equal levels of upregulation in wild type and Myc deficient mice, 0.5 - 3 hours following 14Gy irradiation or cisplatin treatment. Scale bars = 25µm.

**Supplementary Figure 3: Efficient Myc deletion 4 days following Cre induction in *RosaCRE^ERT2^ Myc^fl/fl^* mice. (a-c)** IHC for RFP in colon (a), spleen (b) and thymus (c) of induced *RosaCreER^+^* control and *RosaCreER^+^ Lox-stop-lox RFP* mice. Note RFP positivity in all three tissues indicating successful recombination has occurred. Scale bars = 50µm. **(d)** IHC for Myc in *RosaCRE^ERT2^ Myc^+/+^* and *RosaCRE^ERT2^ Myc^fl/fl^* mice 6h post 14Gy irradiation illustrating deletion of Myc in intestinal crypts following induction with tamoxifen. Scale bars = 50µm.

**Supplementary figure 4: Myc dependent p53 expression in thymus and spleen. (a-c)** IHC for p53 in spleen (a), thymus (b) and colon (c) of induced *RosaCRE^ERT2^ Myc^+/+^* and *RosaCRE^ERT2^ Myc^fl/fl^* mice 6h post 14Gy irradiation illustrating requirement for Myc in p53 expression in spleen and thymus. Scale bars = 50µm.
